# Predictors of Fasting Endogenous Erythritol and Erythronate Concentrations in Humans: Cross-Sectional and Post-Bariatric Surgery Analyses

**DOI:** 10.3390/ijms26199763

**Published:** 2025-10-07

**Authors:** Emilie Flad, Anita Altstädt, Jürgen Drewe, Stefan Gaugler, Christoph Beglinger, Ralph Peterli, Bettina K. Wölnerhanssen, Anne Christin Meyer-Gerspach

**Affiliations:** 1 St. Clara Research Ltd. at St. Claraspital, 4002 Basel, Switzerland; 2 Department of Clinical Research, University of Basel, 4001 Basel, Switzerland; 3 Department of Clinical Pharmacology and Toxicology, University Hospital Basel, 4001 Basel, Switzerland; 4 Institute for Chemistry and Bioanalytics, School of Life Sciences, University of Applied Sciences and Arts Northwestern Switzerland, 4132 Muttenz, Switzerland; 5 Medical Faculty, University of Basel, 4001 Basel, Switzerland

**Keywords:** erythritol, erythronate, endogenous, bariatric surgery

## Abstract

The sugar alcohol erythritol occurs naturally in fruits and fermented foods, is used as a sweetener, and is also endogenously synthesized via the pentose-phosphate pathway and metabolized into erythronate. Untargeted metabolomic studies have associated elevated plasma erythritol and erythronate concentrations with metabolic disorders, while weight loss has been linked to decreased plasma erythritol concentrations. In this trial, two complementary analyses were performed to identify predictors of fasting erythritol and erythronate concentrations across different populations and to assess changes in these metabolites following bariatric surgery-induced weight loss. Fasting plasma samples from 30 lean adolescents, 50 lean adults, and 138 adults with obesity (including 15 who had undergone bariatric surgery) were analyzed to measure erythritol, erythronate, glucose, and insulin concentrations. Across all populations, age but not body mass index (BMI), glucose, or insulin, was a significant predictor of fasting erythritol concentrations. Fasting erythronate concentrations were associated with both age and BMI. Post-surgery, change in BMI but not fasting glucose or insulin was a predictor of changes in fasting erythritol concentrations, while time was the only predictor of changes in fasting erythronate concentrations. Although the metabolic processes regulating the endogenous erythritol and erythronate production remain unclear, our findings suggest that age-related physiological changes may influence fasting concentrations of both erythritol and erythronate.

## 1. Introduction

Erythritol, a sugar alcohol that provides sweetness without contributing calories, has gained attention as an alternative sweetener that promotes satiation without affecting blood glucose and insulin concentrations [1]. It also occurs naturally in some fruits, vegetables, mushrooms, fermented foods and beverages. Erythritol is almost completely absorbed (around 90%) in the small intestine after oral consumption. The majority is excreted unchanged in the urine, with complete excretion occurring within 24 to 78 h [2]. A small fraction of erythritol is further metabolized into erythronate [3]. Additionally, erythritol can be produced endogenously by the human body from glucose via the pentose-phosphate pathway [3]. Several untargeted metabolomic studies have reported elevated plasma erythritol concentrations in individuals with incident central adiposity, obesity, impaired fasting glucose, or type 2 diabetes [3,4]. These elevated concentrations have also been associated with an increased risk of cardiovascular disease [5,6,7,8,9]. Abushamat et al. [7] observed even stronger associations between plasma erythronate concentrations and cardiovascular outcomes than with plasma erythritol concentrations. Of importance, most epidemiological and observational studies have not accounted for the dietary intake of erythritol, making it challenging to differentiate between endogenously produced versus consumed erythritol. However, as most samples in these metabolomics studies were collected prior to the widespread use of erythritol in food, it is more likely that the observed associations reflect endogenous erythritol production. While these observational studies report potential associations, they do not establish causality regarding erythritol’s role in metabolic disorders. Furthermore, the factors driving increased endogenous erythritol production remain unclear.

Interestingly, two interventional trials have reported a decrease in plasma erythritol concentrations following weight loss: First, in the POUNDS Lost trial, participants with overweight or obesity but without diabetes or unstable cardiovascular disease received a calorie-restricted weight-loss program based on dietary counseling with regular group sessions, and assignment to one of four reduced-calorie diets differing in fat and protein content [10]. At baseline, elevated plasma erythritol concentrations were found to be associated with hyperinsulinemia and insulin resistance, as well as a higher 10-year atherosclerotic cardiovascular disease (ASCVD) risk score [11,12]. At both the 6-month and 2-year follow-ups, a greater decrease in plasma erythritol concentrations following weight loss was associated with a greater decrease in fasting insulin concentrations and insulin resistance [11]. Moreover, a greater decrease in plasma erythritol concentrations was associated with greater decreased ASCVD risk scores and improved atherogenic lipid profiles [12]. Second, in the Diabetes Remission Clinical Trial (DiRECT), which included patients with overweight or obesity and type 2 diabetes, a 2-year weight-loss management intervention involving an initial phase of total diet replacement (825–853 kcal/day) followed by structured food reintroduction and long-term weight maintenance support, resulted in decreased concentrations of both erythritol and erythronate [13].

Building on these findings, we hypothesized that fasting erythritol and erythronate concentrations are influenced by age, body mass index (BMI), sex, as well as fasting glucose and insulin concentrations, and are expected to decrease following weight loss induced by bariatric surgery. Two complementary investigations were therefore conducted: a cross-sectional analysis across different age and BMI groups to identify predictors of fasting endogenous erythritol and erythronate concentrations (study 1), and a longitudinal analysis to assess changes in these metabolites following bariatric surgery-induced weight loss (study 2).

## 2. Results

### 2.1. Study 1: Cross-Sectional Analysis of Predictors of Fasting Endogenous Erythritol and Erythronate Across Age and BMI Groups

#### 2.1.1. Clinical Characteristics Across Study Populations

The clinical characteristics of the three study populations are shown in Table 1. Significant differences in age and BMI were observed as defined by the inclusion criteria. Adolescents were significantly younger than both lean adults and adults with obesity (*p* < 0.001). In addition, lean adults were significantly younger than adults with obesity (*p* = 0.011). BMI was similar between lean adolescents and lean adults, while adults with obesity had a significantly higher BMI compared to lean adolescents and lean adults (for both comparisons, *p* < 0.001). Fasting glucose concentrations were similar between lean adolescents and lean adults, while adults with obesity had significantly higher glucose concentrations compared to lean adolescents (*p* < 0.001). Fasting insulin concentrations were similar between lean adolescents and lean adults, while adults with obesity had significantly higher insulin concentrations compared to lean adolescents and lean adults (for both comparisons, *p* < 0.001). Fasting erythritol concentrations were significantly lower in lean adolescents compared to lean adults and adults with obesity (for both comparisons, *p* < 0.001). In addition, lean adults had significantly higher erythritol concentrations compared to adults with obesity (*p* < 0.001). Fasting erythronate concentrations were significantly lower in lean adolescents compared to lean adults and adults with obesity (for both comparisons, *p* < 0.001). In addition, adults with obesity tended to have higher erythronate concentrations compared to lean adults (*p* = 0.055).

#### 2.1.2. Predictors of Fasting Erythritol Concentrations

To identify predictors of fasting erythritol concentrations, a stepwise ordinary regression analysis was performed, including the independent variables sex, age, BMI, fasting glucose, fasting insulin, and fasting erythronate (Table 2). The variables sex (*p* = 0.638), BMI (*p* = 0.468), fasting glucose (*p* = 0.408), and fasting insulin (*p* = 0.290) were removed through backward elimination, resulting in a final model with high overall statistical significance (*p* < 0.001). This model identified age (β = 0.129) and fasting erythronate (β = 0.088) as significant predictors (both *p* < 0.001) of fasting erythritol concentrations.

**Figure 1:**
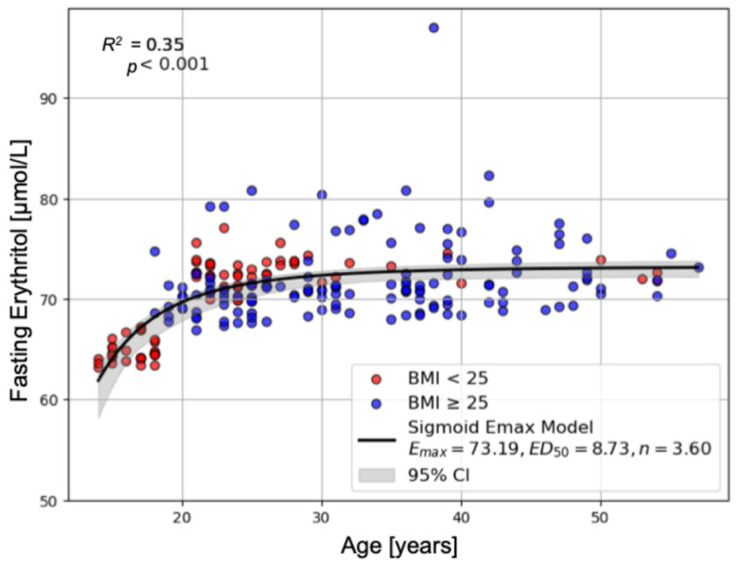
Correlation of Age and Fasting Erythritol Concentrations. This figure shows the non-linear relationship between age and erythritol concentrations that could be best fit by a saturable sigmoid E_max_ model. Data from patients with BMI < 25 are highlighted in red, suggesting that BMI is not influencing the relationship (R^2^ = 0.35, F = 58.466, df1 = 2, df2 = 215, *p* < 0.001).

Increasing age was associated with higher fasting erythritol concentrations. This relationship was non-linear and best described by a saturable model (Figure 1). Higher fasting erythonate concentrations were associated with higher fasting erythritol concentrations.

#### 2.1.3. Predictors of Fasting Erythronate Concentrations

To identify predictors of fasting erythronate concentrations, a stepwise ordinary regression analysis was performed, including the independent variables sex, age, BMI, fasting glucose, fasting insulin, and fasting erythritol (Table 3). The variables sex (*p* = 0.986), fasting glucose (*p* = 0.821), and fasting insulin (*p* = 0.470) were removed through backward elimination, resulting in a final model with high overall statistical significance (*p* < 0.001). This model identified age (β = 0.202), BMI (β = 0.268) and fasting erythritol (β = 0.949) as significant predictors (*p* = 0.027, *p* = 0.010, *p* < 0.001, respectively).

**Figure 2:**
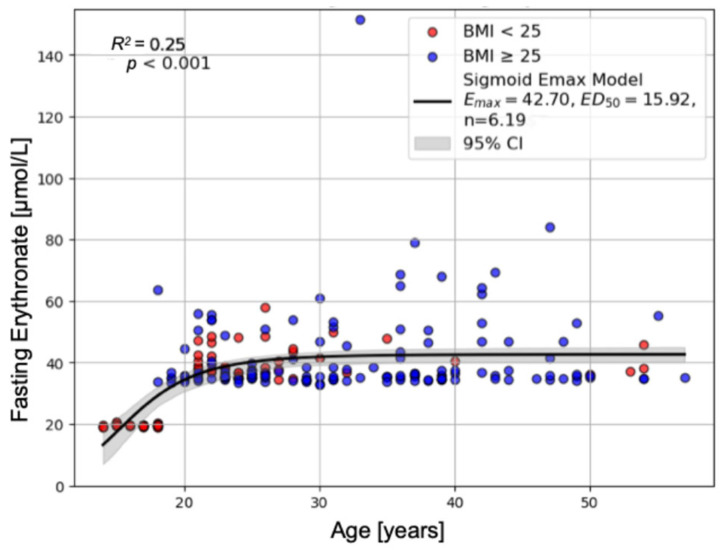
Correlation of Age, BMI, and Fasting Erythronate Concentrations. This figure shows the non-linear relationship between age, BMI, and erythronate concentrations that could be best fit by a saturable sigmoid E_max_ model. Data from patients with BMI < 25 are highlighted in red and with BMI ≥ 25 in blue (R^2^ = 0.25, F = 37.362, df1 = 2, df2 = 215, *p* < 0.001).

Increasing age and a higher BMI were associated with higher fasting plasma erythronate concentrations. This relationship was non-linear and best described by a saturable model (Figure 2). Higher fasting erythritol concentrations were associated with higher fasting erythronate concentrations.

### 2.2. Study 2: Longitudinal Analysis of Changes in Fasting Endogenous Erythritol and Erythronate Following Bariatric Surgery-Induced Weight Loss

#### 2.2.1. Clinical Characteristics Pre- and Post-Bariatric Surgery

The clinical characteristics of pre- and post-surgery are shown in Table 4. Bariatric surgery led to a significant reduction in BMI, which persisted up to 12 months post-surgery (*p* < 0.001). Fasting plasma glucose and insulin concentrations also decreased significantly up to 12 months post-surgery compared to pre-surgery (for all comparisons, *p* ≤ 0.002). No significant changes were observed in fasting erythritol concentrations post-surgery. Fasting erythronate concentrations increased significantly up to 12 months post-surgery (A vs. C: *p* = 0.043 and A vs. D: *p* < 0.001).

#### 2.2.2. Predictors of Changes in Fasting Erythritol Concentrations Post-Surgery

To identify predictors of changes in fasting erythritol concentrations post-surgery, a linear mixed model analysis was performed, including time, age, ΔBMI, Δfasting glucose, Δfasting insulin, and Δfasting erythronate as independent variables (Table 5). The model demonstrated a good fit, as indicated by a log-likelihood value of −58.159. The estimated variances for Group and Time suggest that participants differed somewhat in their baseline concentrations (intercepts) and in how they changed over time, although the relatively large standard errors imply considerable uncertainty. Among the included variables, only ΔBMI (β = 0.354) was significantly associated with changes in fasting erythritol concentrations post-surgery (*p* = 0.025). Specifically, a greater reduction in BMI was associated with a corresponding decrease in fasting erythritol concentrations post-surgery. No significant associations were observed for time, age, Δfasting glucose, Δfasting insulin and Δfasting erythronate post-surgery.

#### 2.2.3. Predictors of Changes in Fasting Erythronate Concentrations Post-Surgery

To identify predictors of changes in fasting erythronate concentrations post-surgery, a linear mixed model analysis was performed, including time, age, ΔBMI, Δfasting glucose, Δfasting insulin, and Δfasting erythritol as independent variables (Table 6). Among the included variables, only time (β = 0.208) was significantly associated with changes in fasting erythronate concentrations post-surgery (*p* < 0.001). Specifically, an increase in time was associated with an increase in fasting erythronate concentrations post-surgery. Otherwise, no associations with other parameters were found.

## 3. Discussion

Plasma erythritol and erythronate originate from endogenous production via the pentose-phosphate pathway or from dietary intake. Their associations with metabolic diseases remain poorly understood, raising questions about the underlying sources of elevated concentrations and whether these metabolites contribute to the development of metabolic diseases or are consequences of those [11,12,13]. Therefore, using fasting plasma samples from studies that excluded dietary erythritol intake, we performed a cross-sectional analysis across different age and BMI groups to identify predictors of fasting endogenous erythritol and erythronate concentrations, as well as a longitudinal analysis to assess changes in these metabolites following bariatric surgery-induced weight loss.

In our cross-sectional analysis, we observed significantly lower fasting erythritol and erythronate concentrations in lean adolescents compared to both lean adults and adults with obesity. Age was significantly associated with fasting erythritol and erythronate concentrations, with higher concentrations observed at increasing ages. In line with our findings, Moon et al. [14] reported a positive correlation between erythritol and erythronate concentrations and age, and Abushamat et al. [7] observed the highest erythritol and erythronate concentrations in the oldest participants with a mean age of 76.7 years. However, in our cohort, the association between age and fasting erythritol and erythronate concentrations appears to be driven by the comparison of adolescents versus adults. The relatively narrow age range within the adult population, with only a small number of participants above 40 years old, limited our ability to evaluate age-related trends across adulthood. During adolescence, numerous metabolic and hormonal adaptations occur, including changes that may reduce insulin sensitivity [15], though, whether these adaptations influence the endogenous erythritol and erythronate production has yet to be elucidated. In adulthood, increasing age is often accompanied by a greater tendency toward metabolic dysregulation, such as hyperglycemia and oxidative stress, which may promote an increased erythritol production via the pentose-phosphate pathway [16]. Thus, while our data highlight clear differences between adolescents and adults, future studies including a broader age spectrum will be important to clarify potential continuous effects of age on erythritol metabolism.

We further identified a positive association between fasting erythritol and erythronate concentrations, which may reflect a secondary effect captured by the regression model. As expected, fasting erythritol emerged as dominant predictor of fasting erythronate concentrations, consistent with its role as the biochemical precursor. In contrast, the reverse association—fasting erythronate predicting fasting erythritol –was unexpected; however, the weak association suggest that fasting erythronate concentrations do not substantially influence its precursor. Thus, fasting erythronate alone cannot explain the variability in fasting erythritol concentrations, and additional covariates, even if not individually significant, may contribute to the observed relationship.

Furthermore, we observed significantly higher fasting erythritol concentrations in lean adults compared to lean adolescents and adults with obesity, but no association was found between fasting erythritol and BMI. However, fasting erythronate concentrations tended to be highest in adults with obesity, and a significant association between fasting erythronate and BMI was found. These findings partially align with the study by Abushamat et al. [7], who reported significantly higher erythritol as well as erythronate concentrations in participants with obesity and diabetes. Hootman et al. [3] observed elevated erythritol concentrations in participants with incident adiposity gain compared to participants with stable adiposity. Taken together, these findings suggest that BMI alone may not be a strong predictor of fasting erythritol and erythronate concentrations.

Hootman et al. [3] additionally reported that erythritol concentrations were higher in participants with higher glycemia (HbA1c concentrations > 5.05%). In our study, no associations were found between fasting erythritol or erythronate concentrations and fasting glucose concentrations. However, it is important to note that we analyzed fasting plasma samples, whereas Hootman et al. [3] did not. Dietary factors, such as sugar intake, may have influenced their findings, as (postprandial) hyperglycemia is linked to increased endogenous erythritol production, and direct dietary intake of erythritol could not be excluded, unlike in our study. In their Mendelian randomization study, Khafagy et al. [17] did not identify a direct association between elevated plasma erythritol concentrations and the risk of diabetes or cardiovascular disease. This finding contrasts with several observational studies, which have observed correlations between higher plasma erythritol concentrations and the presence of diabetes or cardiovascular disease [4,5,6,8,9]. Notably, most of these observational studies involved participants who already had, or were at elevated risk for, diabetes or cardiovascular diseases. In our study, only healthy participants were included, which might explain the contradictory findings.

As observational studies cannot establish causality, interventional studies are needed to identify the potential effects of erythritol on metabolic disorders. Current evidence indicates that erythritol consumption does not impair glycemic control and may exert antioxidative and vascular benefits, particular in participants with diabetes [18,19,20,21,22,23]. Chronic hyperglycemia and associated oxidative stress may upregulate endogenous erythritol and erythronate production through alternative glucose-utilizing pathways, such as the pentose-phosphate pathway, or through the degradation of glycated proteins [7,16,24,25,26,27,28,29,30]. These mechanisms likely reflect an adaptive response aimed at mitigating oxidative damage and elevated glucose concentrations.

Our longitudinal analysis confirmed successful weight loss through bariatric surgery, with a significantly lower BMI up to 12 months post-surgery. Furthermore, the participants showed improved fasting glucose and insulin concentrations post-surgery. Although our model indicated that a greater reduction in BMI was associated with a corresponding decrease in fasting erythritol concentrations, consistent with the findings of the POUNDS Lost and the DiRECT trials, such changes in fasting erythritol were not observed in our cohort. Moreover, the lack of a significant time effect further supports the finding that fasting erythritol concentrations did not change significantly at 3, 6, or 12 months post-surgery. We would have expected a reduction in fasting erythritol concentrations based on the findings of the POUNDS Lost and the DiRECT trials. Given that weight loss following bariatric surgery was substantially greater than that achieved through the weight-loss programs in both interventional trials, greater associated metabolic improvements would also be expected. However, we observed considerable inter-individual variability in changes in fasting erythritol concentrations post-surgery, which may partly account for the absence of a significant overall effect. While some participants showed the expected decrease in fasting erythritol concentrations following weight loss, others exhibited an increase. The present study design does not allow a detailed investigation of the causes of this variability. Although, it is well established that the metabolic effects of bariatric surgery vary between individuals. Furthermore, one of the metabolic changes induced by bariatric surgery seems to be a shift towards the pentose-phosphate pathway to increase the production of NADPH and glutathione, both of which are critical for protecting cells against oxidative stress [31]. This shift may promote endogenous erythritol production which also could have counteracted the decrease in fasting erythritol due to weight loss.

We identified a significant association between changes in fasting erythronate concentrations and time since surgery in the mixed-model analysis, consistent with the gradual rise observed up to 12 months post-surgery in the ANOVA comparison. The underlying mechanism of the changes in fasting erythronate concentrations post-surgery is unknown. Whether this increase is clinically relevant and becomes more pronounced over a longer postoperative period remains to be investigated. Bariatric surgery induces profound early metabolic changes. During the first postoperative year, energy intake is markedly reduced, and rapid weight loss occurs. Subsequently, metabolic adaptation and weight stabilization typically follow [32]. These well-known phases of postoperative adaptation could contribute to the observed time-dependent increase in fasting erythronate concentrations. Larger cohorts with longer follow-up will be required to clarify whether this represents a transient effect of the metabolic adaptation phase or a persistent long-term change.

The findings of our two complementary analyses provide valuable insights into fasting erythritol and erythronate concentrations across a diverse study population and advance the understanding of metabolic factors influencing their endogenous production. Moreover, by excluding participants with regular erythritol intake, we examined endogenously derived erythritol and erythronate—an aspect not considered in untargeted metabolomic studies.

However, several limitations of this study should be considered. First, we included parameters such as sex, age, BMI, fasting glucose, and insulin, that potentially influence the endogenous erythritol and erythronate synthesis, but other potential confounders, including dietary habits, physical activity, hormonal status, and gut microbiota composition were not assessed, even though they may affect metabolism. Future studies should incorporate a broader range of variables to improve prediction models. Second, regular erythritol consumption was assessed by asking participants whether they knowingly consumed erythritol-containing foods or beverages more than once per week. More detailed dietary assessment methods, such as food frequency questionnaires or food diaries, would have provided a more accurate estimate of habitual erythritol consumption. Consequently, we cannot fully exclude that a portion of the measured plasma erythritol originated from exogenous intake. Third, although fasting erythritol and erythronate concentrations were measured using validated gas chromatography–tandem mass spectrometry (GC-MS/MS), differences in analytical sensitivity across laboratories can yield varying absolute concentrations, hindering comparison with values reported elsewhere. No widely accepted reference ranges for plasma erythritol and erythronate concentrations have yet been established. Fourth, the relatively small number of paired plasma samples before and after bariatric surgery limits the statistical power of our longitudinal analysis and may contribute to the observed variability and some conflicting findings. Finally, because samples were collected over an extended period within different studies and by different study personnel, study- or storage-related confounding cannot be completely ruled out. Nevertheless, all personnel were trained and adhered to standardized operation procedures, which should minimize such risks.

## 4. Materials and Methods

### 4.1. Study Population

All fasting plasma samples were obtained between 2012 and 2023 from previous metabolic studies, including dietary intervention and bariatric surgery studies, conducted at the St. Clara Research Ltd. in Basel, Switzerland. All studies were conducted in accordance with the guidelines of the Declaration of Helsinki and national legal and regulatory requirements. The studies were registered at ClinicalTrials.gov: NCT05671965, NCT04713137, NCT04966299, NCT02563847, NCT02821923, NCT02824614, NCT02902224, NCT02902198. Participants provided written informed consent for their samples to be used for scientific research projects. Details about the specific in- and exclusion criteria of the respective studies are provided in the Supplemental Information (Table S1).

#### 4.1.1. Study 1

In this study, we examined 218 fasting plasma samples, including samples from 30 healthy, lean adolescents, 50 healthy, lean adults, and 138 healthy adults with obesity.

All participants were screened for in- and exclusion criteria. For adults, the inclusion criteria were age 18–55 years and BMI 18.0–24.9 kg/m^2^ (lean) or BMI > 30 kg/m^2^ (obese). For adolescents, inclusion criteria were age 14–18 years and BMI in the 15th to 85th percentile, with a minimal body weight of 45 kg. Standard exclusion criteria across all studies included substance abuse; regular intake of medication; relevant acute or chronic infections or diseases (e.g., diabetes or cardiovascular disease); dietary restrictions; pregnancy; and participation in another study with investigational drugs within 30 days prior to or during the study. Additional exclusion criteria, applied in some but not all studies, were smoking, regular intake of pre-/ probiotics, and working night shifts. During the period from which most of our samples were drawn (2012–2016), erythritol was not yet widely used in mainstream foods or beverages in Switzerland, being largely confined to niche sugar-free products. In later studies, when erythritol consumption became more common, regular erythritol consumption (>1 time per week) was an explicit exclusion criterion: During screening, participants were specifically asked about erythritol intake and the use of other sweetener alternatives; participants reporting such products were required to list them in detail, and any individual reporting erythritol-containing products was excluded.

#### 4.1.2. Study 2

In this study, we collected fasting plasma samples of 15 patients pre- and post-bariatric surgery from a previous study.

All participants were screened for in- and exclusion criteria by an interdisciplinary team according to the Swiss guidelines for metabolic bariatric surgery. Inclusion criteria were age 18–55 years and BMI > 35 kg/m^2^. Exclusion criteria were relevant acute/chronic infections or diseases (e.g., diabetes, cardiovascular disease), regular intake of medication, smoking, substance abuse, pregnancy, and dietary restriction. Twelve patients received a laparoscopic Roux-en-Y gastric bypass (LRYGB), and three patients received a laparoscopic sleeve gastrectomy (LSG).

### 4.2. Laboratory Analyses

All blood samples were collected into an ethylenediaminetetraacetic acid (EDTA) precoated tube after an overnight fast from 8 p.m. to 8 a.m. After centrifugation for 10 min at 4 °C at 3000× *g*, the plasma was aliquoted and stored at −80 °C until analysis.

Plasma glucose concentrations were measured using a glucose oxidase method (Roche Diagnostics GmbH, Mannheim, Germany), with an intra- and inter-assay variability below 2.9% and 3.9%, respectively. Plasma insulin concentrations were measured either using an electrochemiluminescence immunoassay (ECLIA) (Roche Diagnostics GmbH, Mannheim, Germany), with an intra- and inter-assay variability below 4.3% and 5.3% and a detection range of 0.4–1000 μIU/mL, or using an enzyme-linked immunosorbent assay (ELISA) (Abnova, Taipei City, Taiwan), with an intra- and inter-assay variability below 8.1% and 8.5% and a detection range of 0–100 μIU/mL.

Plasma erythritol and erythronate concentrations were measured using gas chromatography-tandem mass spectrometry (GC-MS/MS) (FHNW, Muttenz, Switzerland). For this analysis, the samples containing the internal standard xylitol were extracted with methanol/water 7:1 (*v*/*v*), vortexed, and centrifuged at 1400 rpm and 13,300 rpm for five minutes each at room temperature. Subsequently, 70 µL of the supernatant were concentrated for 60 min at 0 mbar on a Gene Vac EZ-2 Elite. Derivatization was performed with methoxyamine for 120 min at 500 rpm and 70 °C, followed by N-Methyl-N-(trimethylsilyl)-trifluoracetamide for 30 min at 500 rpm and 40 °C. Finally, samples were mixed with tert-butylmethyl ether at 1000 rpm prior to GC-MS/MS analysis using helium as carrier gas. Sample derivatization, dilution, and injection was fully automated on a GERSTEL platform (Mülheim a. d. R., Germany). This method was validated according to current ICH guidelines (International Council for Harmonisation of Technical Requirements for Pharmaceuticals for Human Use (ICH). ICH Harmonised Guideline: Validation of Analytical Procedures Q2 (R2) 2023). For plasma erythritol, calibration was linear in the range of 0.1–20 ng/mL with R^2^ = 0.997, and intra-day precision was 0.5% residual standard deviation (RSD) at the mid-calibration point. For plasma erythronate, linearity was 0.1–10 ng/mL with R^2^ = 0.995, and intra-day precision was 0.6% RSD at the mid-calibration point. The lowest calibration point was set as limit of quantification (LOQ) for the method described here. Recovery was 100–111% for erythritol, indicating no significant conversion of erythritol to erythronate during derivatization, nor post-sampling degradation. No carryover was detected under the established MPS wash program. Both derivates showed autosampler stability across 0–96 h. Specificity was confirmed against structurally related compounds, including ribitol, arabitol, mannitol, glucose, and fructose, which confirmed that xylitol is an appropriate internal standard for this study. The peaks were baseline separated (see Supplementary Materials Figure S1). The samples were measured in duplicate.

### 4.3. Statistical Analysis

Statistical analysis of this exploratory study was performed using Python (version 3.9, Python Software Foundation, Wilmington, DE, USA): python package *statsmodels* (version 0.14.4) [33], and scikit-learn SciPy [34,35]. Prior to analysis, missing data were imputed using the median value of the respective parameters. Statistical significance was defined as *p* < 0.05.

#### 4.3.1. Study 1

Differences in parameters (age, BMI, fasting glucose, fasting insulin, fasting erythritol, and fasting erythronate) across the three population groups were assessed using the non-parametric Kruskal-Wallis test (*SciPy* function *kruskal*). When significant, post hoc pairwise comparisons were performed with Dunn’s test for multiple comparisons (using *scikit-learn* functions *posthoc*). Predictors of fasting erythritol and erythronate concentrations were assessed by stepwise ordinary regression analysis, including the independent parameters age, BMI, sex, fasting glucose, and fasting insulin, as well as fasting erythronate or fasting erythritol, respectively, as inputs.

The non-linear relationship between age and effect (fasting erythritol and erythronate concentrations) was evaluated using a sigmoidal E_max_ model [36]:

$$\mathrm{Effect}=\;\frac{E_{max}\times \;{Age}^{n}}{\left({{ED}_{50}}^{n}+{Age}^{n}\right)}$$

where E_max_ is the maximum effect, ED_50_ denotes the age at which 50% of E_max_ is observed, and *n* is the sigmoidicity (Hill) coefficient. Model parameters were estimated by non-linear regression using the *SciPy* function *curve_fit*.

#### 4.3.2. Study 2

Since the amplitudes of the different parameters differed considerably, data were standardized by z-transformation to prevent the overrepresentation of the parameters with the highest amplitude. The dependent variable was not standardized to preserve its original scale. For repeated-measures outcomes (BMI, fasting glucose, fasting insulin, erythritol, erythronate) across time points 0 (baseline), 3, 6, and 12 months post-surgery, we applied repeated-measures ANOVA to test for an overall time effect. Post hoc comparisons of each follow-up time point versus baseline were conducted using paired *t*-tests, with multiplicity adjustment by the Bonferroni–Holm method. This approach provides a conservative control of family-wise error rate, and serves as a practical alternative to Dunnett’s test, which is not applicable in this repeated-measures setting because the post hoc data are not independent. Predictors of changes in fasting erythritol and erythronate concentrations over time were analyzed using linear mixed-effects modelling (LMM) using Restricted Maximum Likelihood Estimation (REML). The dependent variables were the changes in fasting erythritol and erythronate concentrations from pre- to post-surgery (Δerythritol and Δerythronate, respectively). Independent variables included age, time, ΔBMI, Δglucose, Δinsulin, and Δerythronate or Δerythritol, respectively.

## 5. Conclusions

In conclusion, we identified age—but not BMI—as a significant predictor of fasting erythritol concentrations in healthy participants with and without obesity. Fasting erythronate concentrations were associated with both age and BMI. Fasting glucose and insulin concentrations were not predictors of either metabolite. After bariatric surgery, changes in BMI—but not fasting glucose or insulin—predicted changes in fasting erythritol concentrations, whereas time after surgery was the only predictor of changes in fasting erythronate concentrations. Although the metabolic processes regulating the endogenous erythritol and erythronate production remain unclear, our findings suggest that age-related physiological changes may influence fasting concentrations of both erythritol and erythronate.

## Figures and Tables

**Table 1:** Clinical Characteristics Across Study Populations ^1^.

	Lean Adolescents (n = 30) A	Lean Adults (n = 50) B	Adults with Obesity (n = 138) C	*p*-Value ^2^ (Overall)	*p*-Values (Post Hoc)		
					A vs. B	A vs. C	B vs. C
**Sex [number]**	f = 15 m = 15	f = 25 m = 25	f = 87 m = 51				
**Age [years]**	16.37 ± 0.26	27.62 ± 1.22	32.62 ± 0.82	*p* < 0.001	*p* < 0.001	*p* < 0.001	*p* = 0.011
**BMI [kg/m^2^]**	21.01 ± 0.34	22.46 ± 0.23	37.49 ± 0.43	*p* < 0.001	NS	*p* < 0.001	*p* < 0.001
**Fasting Glucose [mmol/L]**	4.89 ± 0.07	4.92 ± 0.06	5.44 ± 0.05	*p* < 0.001	NS	*p* < 0.001	NS
**Fasting Insulin [mIU/L]**	9.00 ± 0.59	8.23 ± 0.79	16.60 ± 0.87	*p* < 0.001	NS	*p* < 0.001	*p* < 0.001
**Fasting Erythritol [µmol/L]**	64.80 ± 0.20	72.66 ± 0.21	71.84 ± 0.34	*p* < 0.001	*p* < 0.001	*p* < 0.001	*p* < 0.001
**Fasting Erythronate [µmol/L]**	19.65 ± 0.08	39.78 ± 0.72	41.41 ± 1.19	*p* < 0.001	*p* < 0.001	*p* < 0.001	*p* = 0.055

^1^ Data shown as mean ± SEM. Significance defined as *p* < 0.05. BMI, body mass index; f, female; m, male; NS, non-significant. BMI categories: underweight < 18.5 kg/m^2^, normal weight 18.5–24.9 kg/m^2^, overweight 25.0–29.9 kg/m^2^, obesity class I 30.0–34.9 kg/m^2^ (n = 48), obesity class II 35–39.9 kg/m^2^ (n = 52), obesity class III ≥ 40 kg/m^2^ (n = 38). Normal reference values: fasting glucose < 5.6 mmol/L; fasting insulin 2–25 mIU/L. ^2^ *p*-values according to the non-parametric Kruskal–Wallis test, with Dunn’s multi-comparison tests.

**Table 2:** Predictors of Fasting Erythritol Concentrations ^1^.

Variable	Estimate	Std. Error	t-Value	*p*-Value	
**(Intercept)**	63.952	0.870	73.495	<0.001	***
**Age**	0.129	0.025	5.151	<0.001	***
**Fasting Erythronate**	0.088	0.019	4.572	<0.001	***
**Model Fit:**					
R^2^ = 0.253, Adj. R^2^ = 0.246					
F(2213) = 36.13, *** *p* < 0.001					

^1^ Significance codes: *** *p* < 0.001. Adj, adjusted; R^2^, coefficient of determination; Std, standard.

**Table 3:** Predictors of Fasting Erythronate Concentrations ^1^.

Variable	Estimate	Std. Error	t-Value	*p-*Value	
**(Intercept)**	−43.848	14.685	−2.986	0.003	**
**Age**	0.202	0.091	2.220	0.027	*
**BMI**	0.268	0.103	2.611	0.010	**
**Fasting Erythritol**	0.949	0.221	4.299	<0.001	***
**Model Fit:**					
R^2^ = 0.222, Adj. R^2^ = 0.211					
F(3214) = 20.32, *p* < 0.001 ***					

^1^ Significance codes: * *p* < 0.05, ** *p* < 0.01, *** *p* < 0.001. Adj, adjusted; BMI, body mass index; R^2^, coefficient of determination; Std, standard.

**Table 4:** Clinical Characteristics Pre- and Post-Bariatric Surgery ^1^.

	Pre- Surgery (n = 15) A	3 Months Post-Surgery (n = 15) B	6 Months Post-Surgery (n = 14) C	12 Months Post-Surgery (n = 13) D	*p*-Value (Overall) ^2^	*p*-Value (Post Hoc)		
						A vs. B	A vs. C	A vs. D
**Sex [number]**	f = 13 m = 2							
**Age [years]**	32.87 ± 2.68							
**BMI [kg/m^2^]**	40.57 ± 1.02	33.60 ± 0.94	29.12 ± 0.90	27.08 ± 1.12	*p* < 0.001	*p* < 0.001	*p* < 0.001	*p* < 0.001
**Fasting Glucose** **[mmol/L]**	5.31 ± 0.14	4.67 ± 0.10	4.60 ± 0.09	4.78 ± 0.10	*p* < 0.001	*p* < 0.001	*p* < 0.001	*p* = 0.001
**Fasting Insulin** **[mIU/L]**	7.61 ± 0.99	3.02 ± 0.29	2.35 ± 0.41	2.82 ± 0.52	*p* < 0.001	*p* < 0.001	*p* = 0.002	*p* = 0.002
**Fasting Erythritol [µmol/L]**	71.44 ± 0.15	71.14 ± 0.20	71.45 ± 0.20	71.35 ± 0.45	NS			
**Fasting Erythronate [µmol/L]**	35.30 ± 0.19	35.55 ± 0.26	36.28 ± 0.30	38.70 ± 0.35	*p* < 0.001	NS	*p* = 0.043	*p* < 0.001

^1^ N = 15; Data shown as mean ± SEM. Significance defined as *p* < 0.05. BMI, body mass index; f, female; m, male, NS, non-significant. Normal reference values: fasting glucose < 5.6 mmol/L; fasting insulin 2–25 mIU/L. ^2^ Repeated measures ANOVA, followed by paired *t*-tests versus baseline with Bonferroni–Holm correction for multiplicity of testing as post hoc test for pairwise comparisons.

**Table 5:** Predictors of Changes in Fasting Erythritol Concentrations Post-Surgery ^1^.

Variable	Estimate	Std. Error	z-Value	*p-*Value	
**(Intercept)**	−0.441	0.470	−0.939	0.348	
**Time**	0.043	0.064	0.671	0.502	
**Age**	−0.101	0.104	−0.970	0.332	
**ΔBMI**	0.354	0.158	2.243	0.025	*
**ΔFasting Glucose**	−0.196	0.110	−1.782	0.075	
**ΔFasting Insulin**	0.113	0.116	0.975	0.329	
**ΔFasting Erythronate**	0.177	0.197	0.900	0.368	
**Group Var**	1.278	1.777			
**Group x Time Cov**	−0.184	0.227			
**Time Var**	0.025	0.031			
**Log-Likelihood**	−58.159				

^1^ Significance codes: * *p* < 0.05. BMI, body mass index; Cov, Covariance; Std, standard; Var, Variation. Mixed linear regression.

**Table 6:** Predictors of Changes in Fasting Erythronate Concentrations Post-Surgery ^1^.

Variable	Estimate	Std. Error	z-Value	*p-*Value	
**(Intercept)**	−1.464	0.276	−5.312	<0.001	***
**Time**	0.208	0.037	5.586	<0.001	***
**Age**	0.212	0.119	1.776	0.076	
**ΔBMI**	0.019	0.162	0.118	0.906	
**ΔFasting Glucose**	−0.117	0.128	−0.918	0.358	
**ΔFasting Insulin**	0.174	0.122	1.431	0.152	
**ΔFasting Erythritol**	−0.075	0.142	−0.524	0.600	
**Group Var**	0.062	0.579			
**Group x Time Cov**	−0.003	0.067			
**Time Var**	0.002	0.010			
**Log-Likelihood**	−51.365				

^1^ Significance codes: *** *p* < 0.001. BMI, body mass index; Cov, Covariance; Std, standard; Var, Variation. Mixed linear regression.

## Data Availability

The raw data supporting the conclusions of this article are not publicly available at this time due to ongoing research but will be made available by the authors upon reasonable request.

## Supplementary Materials

The following supporting information can be downloaded at: https://www.mdpi.com/article/10.3390/ijms26199763/s1.

## Abbreviations

The following abbreviations are used in this manuscript:

ANOVA	Analysis of variance
ASCVD	Atherosclerotic cardiovascular disease
BMI	Body mass index
DiRECT	Diabetes Remission Clinical Trial
ECLIA	Electrochemiluminescence immunoassay
EDTA	Ethylenediaminetetraacetic acid
ELISA	Enzyme-linked immunosorbent assay
GC-MS/MS	Gas chromatography-tandem mass spectrometry
HbA1c	Hemoglobin A1c
LMM	Linear mixed-effects modelling
LOQ	Limit of quantification
LRYGB	Laparoscopic Roux-en-Y gastric bypass
LSG	Laparoscopic sleeve gastrectomy
NADPH	Nicotinamide adenine dinucleotide phosphate hydrogen
NS	Non-significant
REML	Restricted Maximum Likelihood Estimation
RSD	Residual standard deviation
SEM	Standard error of the mean
