# Supplementary material for: Predictors of Fasting Endogenous Erythritol and Erythronate Concentrations in Humans: Cross-Sectional and Post-Bariatric Surgery Analyses
Source: Int J Mol Sci. 2025 Oct 7;26(19):9763. doi: 10.3390/ijms26199763 (PMC12524650; doi:10.3390/ijms26199763)
Supplement: Supplementary file 1 [file ijms-26-09763-s001.zip › ijms-3845676-supplementary.pdf]

International Journal of
Molecular Sciences

MDPI

Supplemental Information

Predictors of Fasting Endogenous Erythritol and Erythronate Concentrations in Humans: Cross-Sectional and Post-Bariatric Surgery Analyses

1. Materials and Methods

1.1. Study Population

For this study, fasting samples from previous metabolic studies, including dietary intervention and bariatric surgery studies, conducted between 2012 and 2023, were analyzed. All inclusion and exclusion criteria for each included study are listed in **Table S1**.

Table S1. In- and Exclusion Criteria of all Included Studies

	NCT05671965	NCT04713137	NCT04966299	NCT02563847	NCT02821923	NCT02824614	NCT02902224	NCT02902198
			Inclusion Criteria					
**Age (yrs)**	18-55	18-55	14-18	18-40	18-55	18-55	18-45	18-45
**BMI (kg/m²)**	19.0-24.9	19.0-24.9	15th – 85th percentile, min. 45 kg	19.0-24.9 (lean) or > 30 (obese)	> 30	> 30	> 35	> 35
**Stable body weight for at least 3 months**	✓	✓	✓	✓	-	-	-	-
Sex	10 females 10 males	10 females 10 males	15 females 15 males	10 females 10 males	18 females 24 males	45 females 18 males	13 females 2 males	6 females 1 male
**Regular sugar consumption (> 25 g/d)**	-	-	✓	-	-	-	-	-
**Right handed**	-	-	-	-	-	-	✓	✓
**Informed consent**	✓	✓	✓	✓	✓	✓	✓	✓
Exclusion Criteria								
**Fructose intolerance**	✓	✓	✓	-	-	-	✓	-
**Food allergies / intolerances**	-	-	-	✓	-	✓	✓	✓
**Pre-existing diet**	✓	✓	✓	✓	-	-	-	-
**Pre-existing erythritol consumption (> 1/week)**	✓	✓	✓	-	-	-	-	-
**Chronic or clinically relevant acute infections/ diseases***	✓	✓	✓	✓	✓	✓	✓	✓
**History of gastrointestinal disorders**	-	-	-	✓	✓	✓	-	-
**Regular intake of medications****	✓	✓	-	✓	✓	✓	✓	✓
**Antibiotics cure within 3 months preceding**	-	-	✓	-	-	✓	-	-
**Regular intake of pro-/prebiotics**	-	-	✓	-	-	✓	-	-
**Pregnancy, breast feeding**	✓	✓	✓	✓	✓	✓	-	-
**Substance abuse**	✓	✓	✓	✓	✓	✓	✓	✓
**Smoking**	-	-		✓	✓	-	✓	✓
**Shift worker**	✓	✓	-	-	-	-	-	-
**Body piercings that cannot be removed**	-	-	-	-	-	-	✓	✓
**Inability to follow procedures due to psychological disorder or language barrier**	-	-	✓	-	✓	✓	-	-
**Participation in another study with investigational drug**	✓	✓	✓	-	✓	✓	-	-

*** Medications:**

- NCT05671965, NCT04713137, NCT02563847, NCT02902224, NCT02902198: all excluded, except oral contraceptives
- NCT04966299: regular medication intake excluded, especially pre- and probiotics, and antibiotics
- NCT02821923: antidepressants, contraceptives, and acid blockers allowed; antihypertensives, lipid-lowering agents, anticoagulants, antiplatelet agents excluded
- NCT02824614: antidepressants and contraceptives allowed; acid blockers, antihypertensives, lipid-lowering agents, anticoagulants, antiplatelet agents, probiotics, prebiotics, antibiotics within the last 3 months excluded

**** Infections / Diseases:**

- NCT05671965, NCT04713137: chronic or clinically relevant acute infections/ diseases excluded
- NCT04966299: all relevant severe acute or chronic diseases excluded
- NCT02821923: known vascular disease, known cardiovascular diseases (coronary heart disease, cardiopathy, arrythmia, pace-maker), diabetes mellitus treated by oral antidiabetics or insulin, arterial hypertension with antihypertensive treatment, dyslipidemia with statin therapy, known chronic hepatic disease (MASH, hepatitis), known renal disease (kidney failure), chronical diseases of the gastrointestinal tract (irritable bowel disease, food intolerance), history of gastrointestinal surgery with major changes to the gastrointestinal tract (removal of stomach, larger portions of the small bowel/colon, any bypass) excluded
- NCT02824614: known cardiovascular diseases (coronary heart disease, cardiopathy, arrythmia, pace-maker), diabetes mellitus treated by oral antidiabetics or insulin, arterial hypertension with antihypertensive treatment, dyslipidemia with statin therapy, known chronic hepatic disease (MASH, hepatitis), known renal disease (kidney failure), chronical diseases of the gastrointestinal tract (irritable bowel disease, food intolerance), history of gastrointestinal surgery with major changes to the gastrointestinal tract (removal of stomach, larger portions of the small bowel/colon, any bypass) excluded
- NCT02563847: medical or psychiatric illness excluded
- NCT02902224, NCT02902198: medical or psychiatric illness, especially diabetes, pace-maker, claustrophobia excluded

1.2. Laboratory Analyses

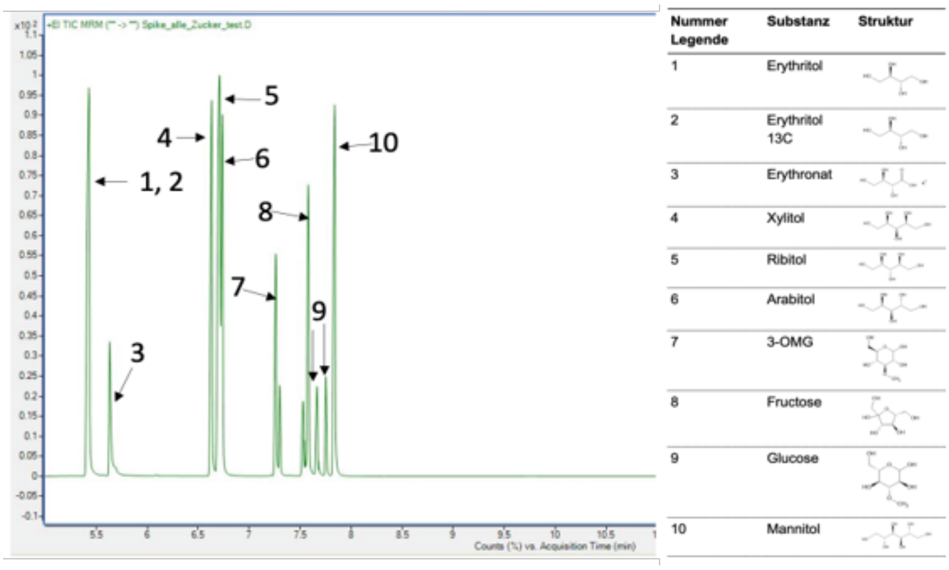

Figure S1: Chromatographic separation and identification of erythritol, erythronate, and structurally related compounds in plasma samples by GC-MS/MS. Representative chromatogram illustrating the baseline separation of erythritol, erythronate, and structurally related compounds. The method demonstrated high specificity with no overlap between peaks. Xylitol served as an effective internal standard. GC-MS/MS, gas chromatography-tandem mass spectrometry.
